# Differences between women and men in prolonged weaning

**DOI:** 10.1186/s12931-024-03002-x

**Published:** 2024-10-08

**Authors:** Evelyn Röser, Julia D. Michels-Zetsche, Hilal Ersöz, Benjamin Neetz, Philipp Höger, Frederik Trinkmann, Michael M. Müller, Laura Klotz, Konstantina Kontogianni, Hauke Winter, Jana Christina Dahlhoff, Sabine Krysa, Felix J. F. Herth, Franziska C. Trudzinski

**Affiliations:** 1 Department of Thoracic Surgery, Thoraxklinik, University Hospital Heidelberg, Roentgenstrasse 1, 69126 Heidelberg, Germany; 2 Translational Lung Research Centre Heidelberg (TLRC-H), German Centre for Lung Research (DZL), Heidelberg, Germany; 3 Department of Pneumology and Critical Care, Translational Lung Research Centre Heidelberg (TLRC-H), Member of the German Centre for Lung Research (DZL), Thoraxklinik, University Hospital Heidelberg, Röntgenstrasse 1, 69126 Heidelberg, Germany

**Keywords:** Prolonged weaning, Sex-specific differences, Weaning outcome, Invasive mechanical ventilation

## Abstract

**Background:**

In recent years, the importance of sex as a factor influencing medical care has received increasing attention in the field of intensive care medicine. The objective of this study was to examine the influence of sex in prolonged weaning.

**Methods:**

A retrospective analysis of patients undergoing prolonged weaning at Thoraxklinik, University Hospital Heidelberg between 12/08 and 12/23 was conducted. Patients with neuromuscular diseases were excluded from the analyses. The risk factors for weaning failure in men and women were identified through stepwise cox-regression analyses.

**Results:**

A total of 785 patients were included, of whom 313 (39.9%) were women. 77.9% of the women and 75.4% of the men were successfully weaned from invasive ventilation. In group comparisons and multivariable analyses, sex was not found to be a risk factor for weaning failure. Cox regression analyses were performed separately for both sexes on the outcome of weaning failure, adjusting for relevant covariates. The results indicated that age ≥ 65 years (HR 2.38, p < 0.001) and the duration of IMV before transfer to the weaning centre (HR 1.01/day, p < 0.001) were independent risk factors in men. In women, however, the duration of IMV before transfer (HR 1.01, p < 0.001), previous non-invasive ventilation (HR 2.9, p 0.005), the presence of critical illness polyneuropathy (HR 1.82; p = 0.040) and delirium (HR 2.50, p = 0.017) were identified as relevant risk factors. In contrast delirium was associated with a favourable weaning outcome in men (HR 0.38, p = 0.020) and nosocomial pneumonia as a reason for prolonged weaning in women (HR 0.43; p = 0.032).

**Conclusion:**

The analyses indicate that there are sex-based differences in the risk factors associated with weaning failure. Further studies, ideally prospective, should confirm these findings to assess whether sex is a factor that should be taken into account to improve weaning outcomes.

**Supplementary Information:**

The online version contains supplementary material available at 10.1186/s12931-024-03002-x.

## Background

The relationship between sex, gender, and outcomes in the intensive care unit (ICU) is complex and multifaceted [1]. About 40% of intubated patients in the ICU are women [2–4] and there is an increasing body of evidence indicating that sex may significantly influence ICU outcomes [1]. Weaning from invasive mechanical ventilation (IMV) is an important part of intensive care medicine that has a significant impact on outcomes. However, potential prognostic differences between mechanically ventilated men and women have been poorly investigated. The few existing studies that have looked at this issue have produced mixed and sometimes conflicting results [5–8]. The risks of weaning failure cover a wide range of different aspects, one of which are upper airway complications. Thille et al. performed a post-hoc analysis of a large clinical trial to assess sex differences in the risk of extubation failure in high-risk ICU patients. They found that men had a significantly higher rate of reintubation within 48 h and was independently associated with an increased risk of reintubation within 7 days [9]. In contrast, the incidence of post-extubation laryngeal oedema is known to be higher in women than in men. This is thought to be due to the fact that the size of the tracheal tube is relatively larger in women compared to the size of the larynx and trachea, which may result in more mechanical trauma and swelling [10, 11]. In prolonged weaning, in addition to airway complications, comorbidities in particular play a decisive role in the success or failure of the process. Cardiac and pulmonary comorbidities are particularly important, with gender-specific differences in chronic lung diseases such as chronic obstructive pulmonary disease (COPD) [12, 13] and cardiac diseases such as heart failure and coronary heart disease being well documented [14, 15]. However, there are few studies examining gender-specific aspects of weaning, with even less data available on prolonged weaning. The aim of our study was to investigate sex aspects in prolonged weaning. We therefore analysed the influence of sex on the success of weaning from IMV in patients undergoing prolonged weaning at the academic weaning centre of the Thoraxklinik, University Hospital Heidelberg.

## Methods

### Study design and participants

This is a single- centre retrospective analysis of prospectively collected data from the German weaning registry WeanNet. WeanNet is an association of specialised weaning centres of the German Society for Pneumology and Respiratory Medicine (DGP) that have undergone a comprehensive quality control process [16]. In addition to personal and structural requirements, the certification includes the inclusion of patients undergoing prolonged weaning in the WeanNet registry. In the current study, the WeanNet data for the weaning centre at the Thoraxklinik, University Hospital Heidelberg from December 2008 to December 2023 were analysed. Patients with neuromuscular diseases were excluded from the analyses because of their presumed low weaning potential. All patients included in the registry met the criteria for prolonged weaning either as defined by the International Consensus Conference (ICC) in April 2005 [17] or the WIND study [18, 19] as weaning group 3 (patients who failed at least three SBT or who require seven days of weaning after the first first separation attempt or SBT), either bevore transfer or during their stay in the ICU or weaning unit at the centre. The data analysis was approved by the Ethics Committee of the University of Heidelberg (No. S-666/2023).

### Weaning process

The weaning process was supported by a multidisciplinary team consisting of pulmonologists/intensivists, respiratory therapists, physiotherapists, specialist nurses, speech therapists, psychologists and social workers. The weaning process was carried out according to a standardised protocol in accordance with the respective current national guidelines [16, 20]. The weaning protocol standard operating procedure of our centre can be found in the supplementary files of the manuscript, see supplementary figures S1 and S2. Once the acute critical illness had resolved and clinical stability was sufficient, patients were transferred to the weaning unit. Firstly, the underlying cause of ventilator dependency was identified, such as respiratory muscle fatigue or weakness, increased respiratory muscle loads, altered respiratory drive or metabolic aspects. The clinical and objective criteria adapted from Boles 2007 [17] were used to assess weaning readiness. A liberal approach was taken to the first SBT, which was performed as early as possible after admission. It was recognised that not all criteria needed to be met for weaning to begin. If weaning readiness was negative, potential causes for this were addressed, such as reducing sedation or treating acute infections. SBTs were performed with low pressure support or on a T-piece/speaking valve, the latter being preferred to the former in tracheostomised patients [21]. Throughout the weaning period, SBTs were extended to enable the reconditioning of the respiratory muscles. Between SBTs, the respiratory muscles were unloaded using assist-control mechanical ventilation in order to avoid a load-capacity imbalance and to allow muscle regeneration. Accompanying measures were secretion management, dynamic hyperinflation therapy, tracheal cannula management, dysphagia management and daily mobilisation. If weaning was unsuccessful, transition to an appropriate health care facility was ensured prior to discharge.

### Weaning outcomes

The weaning outcomes were classified into successful prolonged weaning from IMV without the need for subsequent long-term non-invasive ventilation (NIV), category (3a), successful prolonged weaning from IMV with continuation of NIV (3b) and failed weaning from IMV (3c), differentiated into those with continued IMV in an outpatient setting (3cI) and those who died during the weaning process (3cII) according to the German national guidelines on prolonged weaning [18, 20].

### Statistical analysis

The numerical values are presented as the median, 25th and 75th percentiles, while the categorical values are expressed as numbers and percentages. Group comparisons were analysed using the Mann–Whitney-U-Test for continuous variables and Fisher's exact test for categorical variables. Group comparisons were made between the sexes, but also in terms of weaning success, successful weaning versus weaning failure, defined as the option of discharge with invasive ventilation or death in the weaning centre. The last analysis was performed in the total population, but also separately for men and women.

Values that showed signals either in the total population or in the separate analyses were then further investigated in multivariate analyses, whereby the significance threshold was chosen more liberally than the usual significance threshold (< 0.10 instead of the usual P < 0.05), as our aim was to identify potential predictor variables and not to test a hypothesis. In this context, the scientific plausibility and clinical relevance of the association were also considered. All significant variables were analysed using stepwise cox regression analysis to identify independent risk factors for weaning failure. This was first performed for the entire study population to identify relevant predictive variables. These were then analysed in separate regression analyses for men and women to identify gender differences. The statistical analyses were performed using SPSS version 28 (SPSS Inc., Chicago, Ill., USA).

## Results

### Patient characteristics

From December 2008 to December 2023, a total of 915 patients underwent prolonged weaning at the weaning centre of the Thoraxklinik of the University of Heidelberg. 130 patients were excluded from the analyses due to exclusion criteria. A total of 785 patients, 313 women and 472 men were included in the final analyses. The median age of the patients was 67 years, the mean BMI was 26 kg/m [2] and 14.4% of the patients had a BMI below 20 kg/m [2]. The most common cause of invasive ventilation was COPD exacerbation with 29.7% of cases, followed by pneumonia 20.1% and postoperative acute respiratory insufficiency (ARI) 17.8%. Other recorded causes were: COVID, trauma, restrictive lung disease, sepsis, obesity hypoventilation syndrome (OHS), heart failure and others. Among the comorbidities recorded in the database, cardiopulmonary diseases and obesity were particularly common: COPD in 55.4% of cases, arterial hypertension in 50.1%, heart failure in 38.3% and CHD 29.8% obesity in 28.9%. The patient characteristics stratified for men and women are shown in Table 1**.**

**Table 1:** Patient characteristics by sex

Variables	All N = 785	Females N = 313	Males N = 472	p
Age (years)	67.0 (60.0; 74.0)	67.0 (59.0; 74.0)	68.0 (60.0; 75.0	0.154
Height (cm)	170.0 (160.0; 180.0)	160.0 (160.0; 165.0)	175.0 (170.0; 180.0)	< 0.001
Weight (kg)	80.0 (65.0; 90.0)	80.0 (60.0; 97.3)	75.0 (65.0; 90.0)	0.741
BMI (kg/m^2^)	26.0 (21.5; 32.3)	29.5 (22.9; 37.7)	24.4 (20.8; 29.0)	< 0.001
Smoking status* (%)	12.1/78.3/9.6	13.4/75.1/11.5	11.2/0.5/8.3	0.172
IMV before transfer (days)	18.0 (3.3; 35.0)	17.0 (4.0; 32.8)	19.0 (3.0; 36.0)	0.460
Pre-existing NIV (%)	101 (12.9)	46 (14.7)	55 (11.7)	0.232
Cause of IMV				
COVID (%)	54 (6.9)	22 (7.0)	32 (6.8)	0.887
Pneumonia (%)	158 (20.1)	50 (16.0)	108 (22.9)	0.018
AECOPD (%)	233 (29.7)	100 (31.9)	133 (28.2)	0.265
ARDS (%)	74 (9.4)	28 (8.9)	46 (9.7)	0.803
Trauma (%)	7 (0.9)	2 (0.6)	5 (1.1)	0.709
Postoperative ARI (%)	140 (17.8)	50 (16.0)	90 (19.1)	0.295
Restrictive lung disease (%)	27 (3.4)	8 (2.6)	19 (4.0)	0.321
Sepsis (%)	34 (4.3)	14(4.5)	20 (4.2)	0.860
OHS (%)	36 (4.6)	25 (8.0)	11 (2.3)	< 0.001
Heart failure (%)	33 (4.2)	16 (5.1)	17 (3.6)	0.364
Others (%)	43 (5.5)	20 (6.4)	23 (4.9)	0.424
Comorbidities				
None (%)	19 (2.4)	12 (13.8)	7 (1.5)	0.055
Heart failure (%)	301 (38.3)	117 (37.4)	184 (39.0)	0.708
ILD (%)	30 (3.8)	8 (2.6)	22 (4.7)	0.182
COPD (%)	435 (55.4)	172 (55.0)	263 (55.7)	0.883
Restrictive lung disease (%)	115 (14.6)	36 (11.5)	79 (16.7)	0.050
Arterial hypertension (%)	393 (50.1)	154 (49.2)	239 (50.6)	0.716
CIP (%)	187 (23.8)	74 (23.6)	113 (23.9)	0.932
Obesity (%)	227 (28.9)	125 (39.9)	102 (21.6)	< 0.001
CAD (%)	234 (29.8)	75 (24.0)	159 (33.7)	0.004
Renal failure (%)	173 (22.0)	61 (19.5)	112 (23.7)	0.187
Diabetes mellitus (%)	217 (27.6)	95 (30.4)	122 (25.8)	0.192
Immunosuppression (%)	25 (3.2)	9 (2.9)	16 (3.4)	0.836
Oncological diseases (%)	96 (12.2)	21 (8.6)	69 (14.6)	0.014
PH (%)	53 (6.8)	29 (9.3)	24 (5.1)	0.029
Delirium (%)	111 (14.1)	32 (10.2)	79 (16.7)	0.012
Nosocomial pneumonia (%)	189 (24.1)	67 (21.4)	122 (25.8)	0.173

^*^(never/former/current)

*BMI* body mass index, *IMV* invasive mechanical ventilation, *NIV* non-invasive ventilation, *AECOPD* acute exacerbated chronic obstructive pulmonary disease, *ARDS* acute respiratory distress syndrome, *ARI* acute respiratory insufficiency, *OHS* obesity hypoventilation syndrome, *ILD* interstitial lung disease, *COPD* chronic obstructive pulmonary disease, *CIP* critical illness associated polyneuropathy, *CAD* coronary artery disease, *PH* pulmonary hypertension. The group comparisons were carried out using the Fisher’s exact test or Mann–Whitney U-tests, p values < 0.05 were considered statistically significant

### Differences between men and women

Compared to the male patients, females were smaller (160 vs. 175 cm; p < 0.001), but had a significantly higher BMI (29.5 vs. 24.4 kg/m [2]; p < 0.001). The duration of IMV before transfer to the weaning centre did not differ between the sexes, but men had a longer hospital stay in the weaning centre than women (38 vs 32 days; < 0.001). With regard to the causes of IMV, pneumonia was more frequently reported as a reason for IMV in men (16.0% vs. 22.9%; p = 0.018) and obesity hypoventilation syndrome (OHS) in women (8.0% vs. 2.3%; p < 0.001). But there were no differences between the sexes for the other causes. In terms of comorbidities, there were differences in the diagnosis of obesity in 39.9% of women and 21.6% of men (p < 0.001), in line with the recorded BMI values, and women were more likely to have pulmonary hypertension. Men were more likely to have oncological diagnoses (14.6 vs. 8.6%; p = 0.014) and delirium (16.7 vs. 10.2%; p = 0.012), see Table 1**.**

### Weaning outcomes for men and women.

**Fig. 1:**
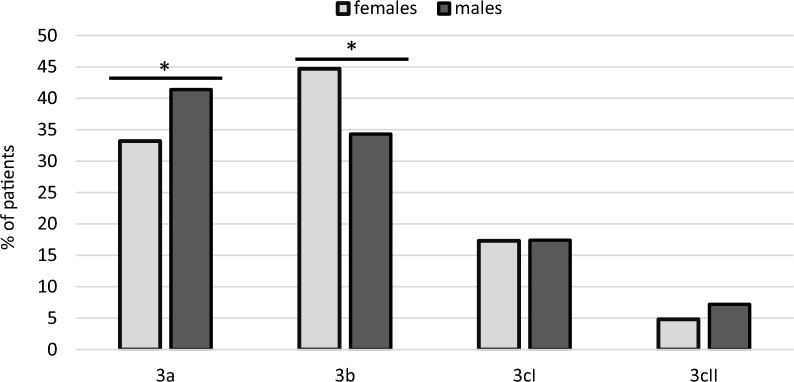
Weaning outcomes for men and women N = 785. According to German guidelines, patients were categorised as follows: category 3a: successful prolonged weaning from mechanical ventilation without the need for subsequent long-term non-invasive ventilation (NIV); category 3b: successful prolonged weaning from mechanical ventilation with the continuation of NIV and from mechanical ventilation; category 3cI: failed weaning, patients who continued to receive mechanical ventilation as an outpatient; and category 3cII: failed weaning, patients who died during weaning. The figure shows the number of patients in the individual categories as a percentage. *p < 0.005

Overall, 244 (77.9%) women and 356 (75.4%) men were successfully weaned from invasive ventilation (p = 0.440). Those women who were successfully weaned were more likely than men to require non-invasive ventilation (category 3b; 44.7% vs. 34.3%; p = 0.004) and men were more likely to be discharged without NIV (category 3a; 41.1% vs. 33.2%; p = 0.029). There were no significant sex differences in discharge with IMV (17.3% of women and 17.4% of men; p = 1,000) and death in the weaning centre (4.8% of women and 7.2% of men; p = 0.179). The weaning outcomes for men and women are shown in Fig. 1**.**

### Predictors of weaning failure in men and women

In the group comparisons, older age and BMI ≤ 20 kg/m [2] were identified as risk factors for weaning failure in men but not in women. The duration of prior IMV was a relevant factor only in women. Women and Men with unsuccessful weaning also had longer stays in the weaning unit (42 vs. 31.5 days; p = 0.002 and 47 vs. 36 days; p = 0.009). Women with unsuccessful weaning were more likely to have critical illness polyneuropathy (40.6 vs. 18.9%; p < 0.001) and delirium (17.4 vs. 8.2%; p = 0.040), whereas there were no disadvantageous group differences in these comorbidities in men see Table 2.

**Table 2:** Successful weaning versus weaning failure by sex

Variables	Females			Males		
	Successful weaning N = 244	Weaning failure N = 69	p	Successful weaning N = 356	Weaning failure N = 116	p
Age (years)	66.5 (59.0; 74.0)	68.5 (59.3; 77.0)	0.314	66.0 (59.0; 73.0)	72.0 (65.0; 77.0)	< 0.001
Height (cm)	160 (160; 165)	160 (160; 165)	0.387	175 (170; 180)	175 (170; 180)	0.362
Weight (kg)	80.0 (60.0; 98.5)	80.0 (58.8; 96.3)	0.219	80.0 (65.0; 90.0)	75.0 (60.0; 90.0)	**0.054**
BMI (kg/m2)	29.7 (23.0; 37.6)	29.5 (21.9; 39.1)	0.342	24.7 (21.2; 29.3)	24.2 (19.6; 27.8)	< 0.001
Smoking status* (%)	14.3/73.4/12.3	10.1/81.2/8.7	0.417	10.7/80.3/9.0	12.9/81.0/86.0	0.515
IMV before transfer (days)	14.0 (1.8; 27.3)	31.0 (15.3; 81.5)	0.022	19.0 (4.0; 35.0)	18.0 (0.0; 44.0)	0.786
Pre-existing NIV (%)	29 (11.9)	17 (24.6)	0.012	36 (10.1)	19 (16.4)	0.094
Cause of IMV						
COVID (%)	19 (7.8)	3 (4.3)	0.430	28 (7.9)	4 (3.4)	0.135
Pneumonia (%)	41 (16.8)	9 (13.0)	0.577	85 (23.9)	23 (19.8)	0.445
AECOPD (%)	78 (32.0)	22 (31.9)	1.000	98 (27.5)	35 (30.2)	0.635
ARDS (%)	26 (10.7)	2 (2.9)	0.055	36 (10.1)	10 (8.6)	0.721
Trauma (%)	2 (0.8)	0 (0.0)	1.000	4 (1.1)	1 (0.9)	1.000
Postoperative ARI (%)	40 (16.4)	10 (14.5)	0.853	65 (18.3)	25 (21.6)	0.418
Restrictive lung disease (%)	8 (2.0)	5 (4.3)	0.381	13 (3.7)	6 (5.2)	0.428
Sepsis (%)	10 (4.1)	4 (5.8)	0.518	16 (4.5)	4 (3.4)	0.793
OHS (%)	21 (8.6)	4 (5.8)	0.616	10 (2.8)	1 (0.9)	0.308
Heart failure (%)	10 (4.1)	6 (8.7)	0.131	13 (3.7)	4 (3.4)	1.000
Others (%)	11 (4.5)	9 (13.0)	0.021	16 (4.5)	7 (6.0)	0.467
Comorbidities						
None (%)	9 (3.7)	3 (4.3)	0.731	5 (1.4)	2 (1.7)	0.683
Heart failure (%)	89 (36.5)	28 (40.6)	0.574	130 (36.5)	54 (46.6)	0.062
ILD (%)	6 (2.5)	2 (2.9)	1.000	18 (5.1)	4 (3.4)	0.616
COPD (%)	137 (56.1)	35 (50.7)	0.494	192 (53.9)	71 (61.2)	0.197
Restrictive lung disease (%)	24 (9.8)	12 (17.4)	0.090	53 (14.9)	26 (22.4)	0.064
Arterial hypertension (%)	125 (51.2)	29 (42.0)	0.220	181 (50.8)	58 (50.0)	0.915
CIP (%)	46 (18.9)	28 (40.6)	< 0.001	85 (23.9)	28 (24.1)	1.000
Obesity (%)	100 (41.0)	25 (36.2)	0.491	79 (22.2)	23 (19.8)	0.697
CAD (%)	56 (23.0)	19 (27.5)	0.428	120 (33.7)	39 (33.6)	1.000
Renal failure (%)	43 (17.6)	18 (26.1)	0.124	78 (21.9)	34 (29.3)	0.131
Diabetes mellitus (%)	68 (27.9)	27 (39.1)	0.077	89 (25.0)	33 (28.4)	0.466
Immunosuppression (%)	7 (2.9)	2 (2.9)	1.000	12 (3.4)	4 (3.4)	1.000
Oncological diseases (%)	19 (7.8)	8 (11.6)	0.334	48 (13.5)	21 (18.1)	0.228
PH (%)	20 (8.2)	9 (13.0)	0.241	21 (5.9)	3 (2.6)	0.223
Delirium (%)	20 (8.2)	12 (17.4)	0.040	62 (17.4)	17 (14.7)	0.568
Nosocomial pneumonia (%)	59 (24.2)	8 (11.6)	0.030	93 (26.1)	29 (25.0)	0.903

^*^(never/former/current)

*BMI* body mass index, *IMV* invasive mechanical ventilation, *NIV* non-invasive ventilation, *AECOPD* acute exacerbated chronic obstructive pulmonary disease, *ARDS* acute respiratory distress syndrome, *ARI* acute respiratory insufficiency, *OHS* obesity hypoventilation syndrome, *ILD* interstitial lung disease, *COPD* chronic obstructive pulmonary disease, *CIP* critical illness associated polyneuropathy, *CAD* coronary artery disease, *PH* pulmonary hypertension. The group comparisons were carried out using the Fisher's exact test or Mann–Whitney U-tests, p values < 0.05 were considered statistically significant

### Independent predictors of weaning failure in men and women

**Fig. 2:**
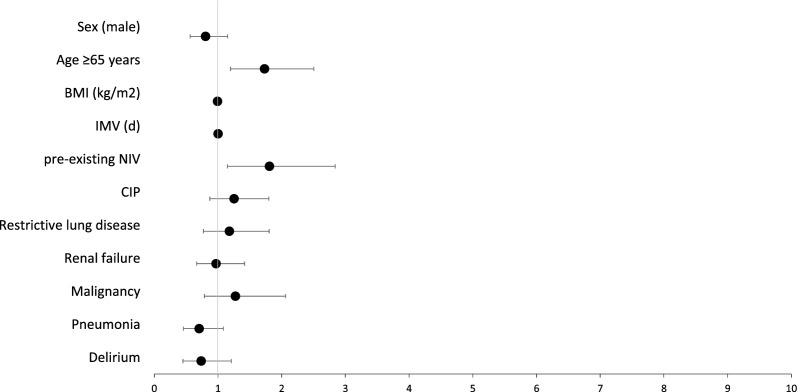
Results of Cox regression analysis for Risk factors for weaning failure in the study population N = 785. The figure shows the results of the Cox regression analysis with weaning failure as outcome variable. The plot shows the Hazard Ratios with their corresponding 95% confidence intervals corresponding to the numerical values given in supplementary Table S1. *IMV* Invasive mechanical ventilation before transfer to the centre, *CIP* Critical illness polyneuropathy, *BMI* body mass index, *NIV* non-invasive ventilation

The cox regression analyses performed with the outcome parameter weaning failure, revealed that sex had no effect on the outcome variable in the population. In the overall study population, age ≥ 65 years (HR 1.73, 95% CI 1.20–2.50; p < 0.005), IMV before transfer to the weaning centre (HR 0.001, 95% CI 1.00–1.00; < 0.001), pre-existing NIV (HR 1.81, 95% CI 1.15–2.84; p = 0.010), were relevant independent risk factors for weaning failure. Please refer to Fig. 2 and the supplementary Tables S1 for further details.

**Fig. 3:**
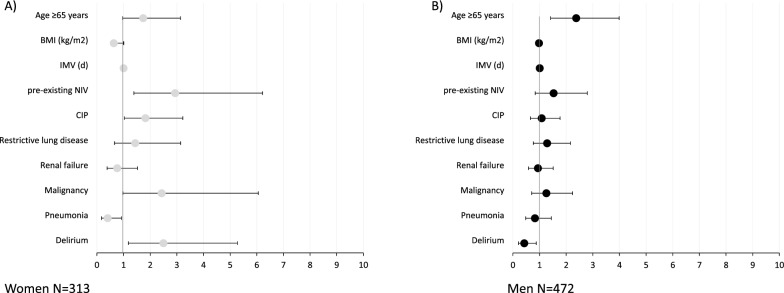
Results of Cox regression analysis for Risk factors for weaning failure women and in men. The figure shows the results of the Cox regression analysis with weaning failure as the outcome variable, which were carried out separately for women (**A**) and men (**B**). The plot shows the Hazard Ratios with their corresponding 95% confidence intervals corresponding to the numerical values given in supplementary Table S 2 and 3. IMV = Invasive mechanical ventilation before transfer to the centre, *CIP* Critical illness polyneuropathy, *BMI* body mass index, *NIV* non-invasive ventilation

Subsequently, regression analyses were conducted on the same set of covariates, with the results stratified by sex. In females, the duration of IMV prior to transfer (HR 1.01, 95% CI 1.00–1.01; p < 0.001), CIP (HR 1.82, 95% CI 1.03–3.23; p = 0.040) and delirium (HR 2.50, 95% CI 1.18–5.27; p = 0.017) were identified as relevant risk factors, while pneumonia was identified as a favourable factor (HR 0.40, 95% CI 0.18–0.93; p = 0.032). For men, only age ≥ 65 years (HR 2.38, 95% CI 1.42–3.99; p < 0.001) and IMV prior to transfer (HR 1.01, 95% CI 1.01–1.01/; p < 0.001 were identified as relevant risk factors. However, it is noteworthy that delirium had the opposite effect for men and proved to be a favourable factor for weaning success in the subgroup of men (HR 0.21, 95% CI 0.21–0.88; p = 0.020). Please refer to Fig. 3 and the supplementary Table S2 and 3 for further details.

## Discussion

The objective of this study was to examine the influence of sex on the weaning outcome in prolonged weaning. The main results are that sex did not influence the outcome of weaning; but factors associated with prolonged weaning failure were not the same among males and females. The fact that around 40% of the patients in our group were also female fits in surprisingly well with the general prevalence of women in the intensive care unit [2–4].

Another important finding of the study is that the risk factors for prolonged weaning differ significantly between men and women. In some cases, there were even opposing effects, with delirium being associated with unfavourable outcomes in women and favourable outcomes in men.

In the general population over the age of 65, the duration of prior invasive mechanical ventilation (IMV) and the presence of pre-existing non-invasive ventilation (NIV) were identified as independent predictors of weaning failure. Separate Cox regression analyses for men and women revealed differing influential factors between the sexes. In women, the duration of invasive ventilation before transfer to the weaning center, pre-existing NIV, and the co-occurrence of critical illness polyneuropathy or delirium emerged as independent risk factors for weaning failure. In men, however, only age and the duration of invasive ventilation prior to transfer were significant. Interestingly, the effect of delirium differed between sexes; in men, a diagnosis of delirium was linked to a more favorable prognosis regarding weaning failure. Additionally, in women, the presence of nosocomial pneumonia as the cause of prolonged IMV was associated with a better weaning outcome. The reasons behind these sex-based differences remain speculative. Delirium is an important predictor of unfavourable clinical outcomes in the ICU, including prolonged IMV. Jeon et al. studied delirium in 393 patients receiving MV who underwent a spontaneous breathing trial. They found that 40.7% of the patients were diagnosed with delirium on the day of the first SBT. After adjustment for potential confounders, delirium was significantly associated with difficult weaning but not with prolonged weaning [22]. Similarly, in prolonged weaning, delirium may lead to a greater need for sedation and therefore a longer duration of mechanical ventilation, not necessarily associated with weaning failure. In our population of patients undergoing prolonged weaning, delirium was diagnosed more frequently in men than in women, with the diagnosis having a favourable effect on weaning success in men and an unfavourable effect in women. The observed differences may be explained by a gender bias in delirium diagnosis. It is possible that delirium was diagnosed earlier but less severe in men, which could be explained by the fact that men have a different delirium subtypes than women. A study of 406 adults, non-demented patients with delirium showed that men had higher scores for motor agitation and affective lability in the subscores of the Delirium Rating Scale-Revised-98 (DRS-R-98), while women were more frequently diagnosed with hypoactive delirium [23]. In particular, the hypoactive subtype of delirium presents a diagnostic and clinical challenge. As the symptoms of hypoactive delirium can overlap with those of dementia and depression, correct diagnostic differentiation can be difficult. Without timely diagnosis and treatment, hypoactive delirium can persist for several weeks [24]. A connection with weaning failure is therefore quite conceivable.

The association between pneumonia and weaning success, and the fact that it was only observed in women, was unexpected to us. Pneumonia acquired in the ICU usually has a negative impact on morbidity, prolonged length of stay and duration of MV treatment in patients with ventilator- associated pneumonia [25]. In our particular patient population, which consists mainly of patients who had to be transferred to a specialised centre for prolonged weaning, the diagnosis must be evaluated against the background of other causes of mechanical ventilation and comorbidities in this patient population. Compared to the usual chronic cardiopulmonary conditions of these patients, pneumonia is a reversible and treatable cause of prolonged ventilation that does not necessarily lead to an increased risk of weaning failure. The former mentioned effects of nosocomial pneumonia in males may be outweighed by the problems and complications of pneumonia. Known sex differences could play a role here as men and women differ in their innate immune responses. Men are more likely than women to develop infectious diseases, with an average annual relative risk of 1.3 and an almost 1.6 times higher risk of hospitalisation for sepsis [1]. Furthermore there are relevant sex-specific differences in the lungs: even when taking body size and lung volume into account, women have smaller airway lumina [26] and a diaphragm that is approximately 9% shorter than men [1]. It can therefore be assumed that they have a lower functional reserve of the respiratory pump than men due to the lower power at higher loads and that the duration of previous ventilation or the effects of CIP have a correspondingly greater effect on weaning success in women compared to men.

The observation that age was a relevant factor for weaning failure only in men suggests that selection effects regarding previous intensive care therapy may also have played a role in this subgroup. It is known that women less likely receive mechanical ventilation or renal replacement therapy than men [27]. In addition to the medical decision to utilise such therapies, socio-cultural factors such as socio-economic status, formal education and patient preferences also play a role in access to intensive care therapies [1]. Women are more likely to restrict life-sustaining therapies, especially in older age groups or if they are divorced or widowed [28].

### Limitations

Although the data were prospectively entered into the WeanNet registry, this study is a retrospective data analysis of a single centre and should be interpreted accordingly. There is a lack of information regarding the timing and content of the comorbidities mentioned. It is therefore not possible to determine the specific subtype, the point of onset or the duration of delirium or give a definition of what was considered immunosuppression. The same applies to nosocomial pneumonia. It should be emphasised that the diagnosis of VAP in the ICU is often clinically challenging, and again there is no chronological reference as to whether the pneumonia occurred immediately after acute therapy and was ultimately the cause of the patient's prolonged weaning, or whether the problem first occurred in the weaning centre.

## Conclusion

Several sex-specific differences were observed in the process of prolonged weaning. While baseline characteristics and overall weaning outcomes showed minimal variation between genders, the factors linked to weaning failure in women differed significantly from those in men. A key factor contributing to these differences is likely the interaction of biological and immunological mechanisms that may influence outcomes. Additionally, the role of systemic and implicit biases, along with cognitive errors related to both sex and gender bias from patients and healthcare professionals, is less understood but may also contribute. These results raise the question of whether patient sex should be factored into therapeutic decision-making during the weaning process. However, given the retrospective nature of this study, these findings should be interpreted cautiously, and further prospective research is needed to confirm and refine these insights.

## Supplementary Information

Supplementary file 1.

Supplementary file 2.

## Data Availability

The datasets used and analysed during the current study are available from the corresponding author on reasonable request.

## Abbreviations

ABG: Arterial blood gas
AECOPD: Acute exacerbated chronic obstructive pulmonary disease
ARDS: Acute respiratory distress syndrome
ARI: Acute respiratory insufficiency
AUC: Area under the curve
B: Unstandardised estimate
BMI: Body mass index
CAD: Coronary artery disease
CI: Confidence interval
CIM: Critical illness associated myopathy
CIP: Critical illness associated polyneuropathy
COPD: Chronic obstructive pulmonary disease
F_i_O_2_: Inspiratory fraction of oxygen
HCO_3_^−^: Bicarbonate
ICC: International Consensus Conference
ICU: Intensive care unit
ILD: Interstitial lung disease
IMV: Invasive mechanical ventilation
N: Number
NIV: Non-invasive ventilation
NMD: Neuromuscular disease
O_2_: Oxygen
OHS: Obesity hypoventilation syndrome
HR: Hazard ratio
P: Probability
PEEP: Positive end expiratory pressure
pH: Potential of hydrogen
ROC: Receiver operating characteristic
SaO_2_: Arterial saturation of oxygen
SBT: Spontaneous breathing trial
SE: Standard error
VWU: Ventilator weaning unit
