# Supplementary material for: Differences between women and men in prolonged weaning
Source: Respir Res. 2024 Oct 8;25:363. doi: 10.1186/s12931-024-03002-x (PMC11460207; doi:10.1186/s12931-024-03002-x)
Supplement: Supplementary file 1 [file 12931_2024_3002_MOESM1_ESM.docx]

| **Variable** | **HR** | **95%CI of coefficient B** | | **P-value** |
| --- | --- | --- | --- | --- |
|  |  | **Lower** | **Upper** |  |
| Sex (male) | 0.81 | 0.56 | 1.15 | 0.236 |
| Age ≥65 years | 1.73 | 1.20 | 2.50 | 0.004 |
| BMI (kg/m^2^) | 0.99 | 0.98 | 1.01 | 0.440 |
| IMV (d) | 1.00 | 1.00 | 1.00 | <0.001 |
| pre-existing NIV | 1.81 | 1.15 | 2.84 | 0.010 |
| Critical illness polyneuropathy | 1.25 | 0.87 | 1.80 | 0.221 |
| Restrictive lung disease | 1.18 | 0.77 | 1.80 | 0.444 |
| Renal failure | 0.97 | 0.66 | 1.42 | 0.875 |
| Malignancy | 1.27 | 0.79 | 2.06 | 0.324 |
| Pneumonia | 0.70 | 0.46 | 1.08 | 0.111 |
| Delirium | 0.74 | 0.45 | 1.21 | 0.225 |

**Table S1 Cox regression analysis to assess the independent risk factors for weaning failure in the entire study population**

The Table shows the results of the Cox regression analysis with weaning failure as outcome variable. HR = Hazard Ratio, BMI = body mass index, NIV= non-invasive ventilation, N=785.

| **Variable** | **HR** | **95%CI of coefficient B** | | **P-value** |
| --- | --- | --- | --- | --- |
|  |  | **Lower** | **Upper** |  |
| Age ≥65 years | 1.74 | 0.96 | 3.14 | 0.067 |
| BMI (kg/m^2^) | 1.00 | 0.99 | 1.01 | 0.632 |
| IMV (d) | 1.01 | 1.00 | 1.01 | <0.001 |
| pre-existing NIV | 2.94 | 1.39 | 6.22 | 0.005 |
| Critical illness polyneuropathy | 1.82 | 1.03 | 3.23 | 0.040 |
| Restrictive lung disease | 1.44 | 0.66 | 3.14 | 0.362 |
| Renal failure | 0.76 | 0.38 | 1.52 | 0.443 |
| Malignancy | 2.43 | 0.98 | 6.06 | 0.057 |
| Pneumonia | 0.40 | 0.18 | 0.93 | 0.032 |
| Delirium | 2.50 | 1.18 | 5.27 | 0.017 |

**Table S2 Cox regression analysis to assess the independent risk factors for cessation in women**

The Table shows the results of the binary logistic regression analysis with weaning failure as outcome variable, which were carried out separately for women. HR = Hazard Ratio, BMI = body mass index, NIV= non-invasive ventilation, N=785.

| **Variable** | **HR** | **95%CI of coefficient B** | | **P-value** |
| --- | --- | --- | --- | --- |
|  |  | **Lower** | **Upper** |  |
| Age ≥65 years | 2.38 | 1.42 | 3.99 | <0.001 |
| BMI (kg/m^2^) | 0.98 | 0.95 | 1.02 | 0.267 |
| IMV (d) | 1.01 | 1.01 | 1.01 | <0.001 |
| pre-existing NIV | 1.53 | 0.84 | 2.79 | 0.169 |
| Critical illness polyneuropathy | 1.08 | 0.66 | 1.77 | 0.757 |
| Restrictive lung disease | 1.29 | 0.76 | 2.16 | 0.343 |
| Renal failure | 0.94 | 0.59 | 1.51 | 0.805 |
| Malignancy | 1.26 | 0.71 | 2.24 | 0.439 |
| Pneumonia | 0.83 | 0.48 | 1.44 | 0.508 |
| Delirium | 0.43 | 0.21 | 0.88 | 0.020 |

**Table S3 Risk factors for weaning failure in men**

The Table shows the results of the binary logistic regression analysis with weaning failure as outcome variable, which were carried out separately for men. HR = Hazard Ratio, BMI = body mass index, NIV= non-invasive ventilation
