# Supplementary material for: Differences between women and men in prolonged weaning
Source: Respir Res. 2024 Oct 8;25:363. doi: 10.1186/s12931-024-03002-x (PMC11460207; doi:10.1186/s12931-024-03002-x)
Supplement: Supplementary file 2 [file 12931_2024_3002_MOESM2_ESM.pptx]

## Slide 1
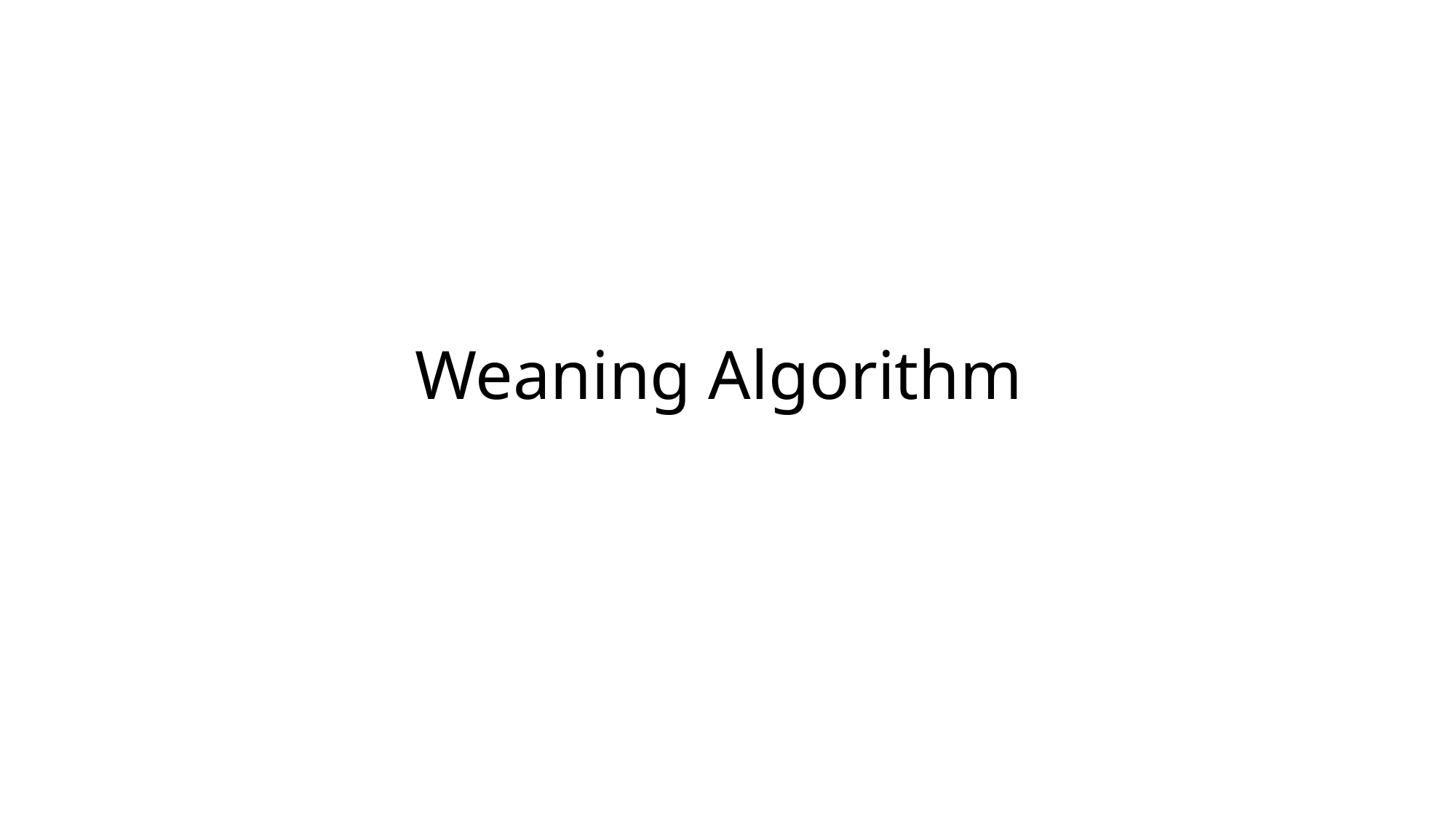

# Weaning Algorithm

## Slide 2
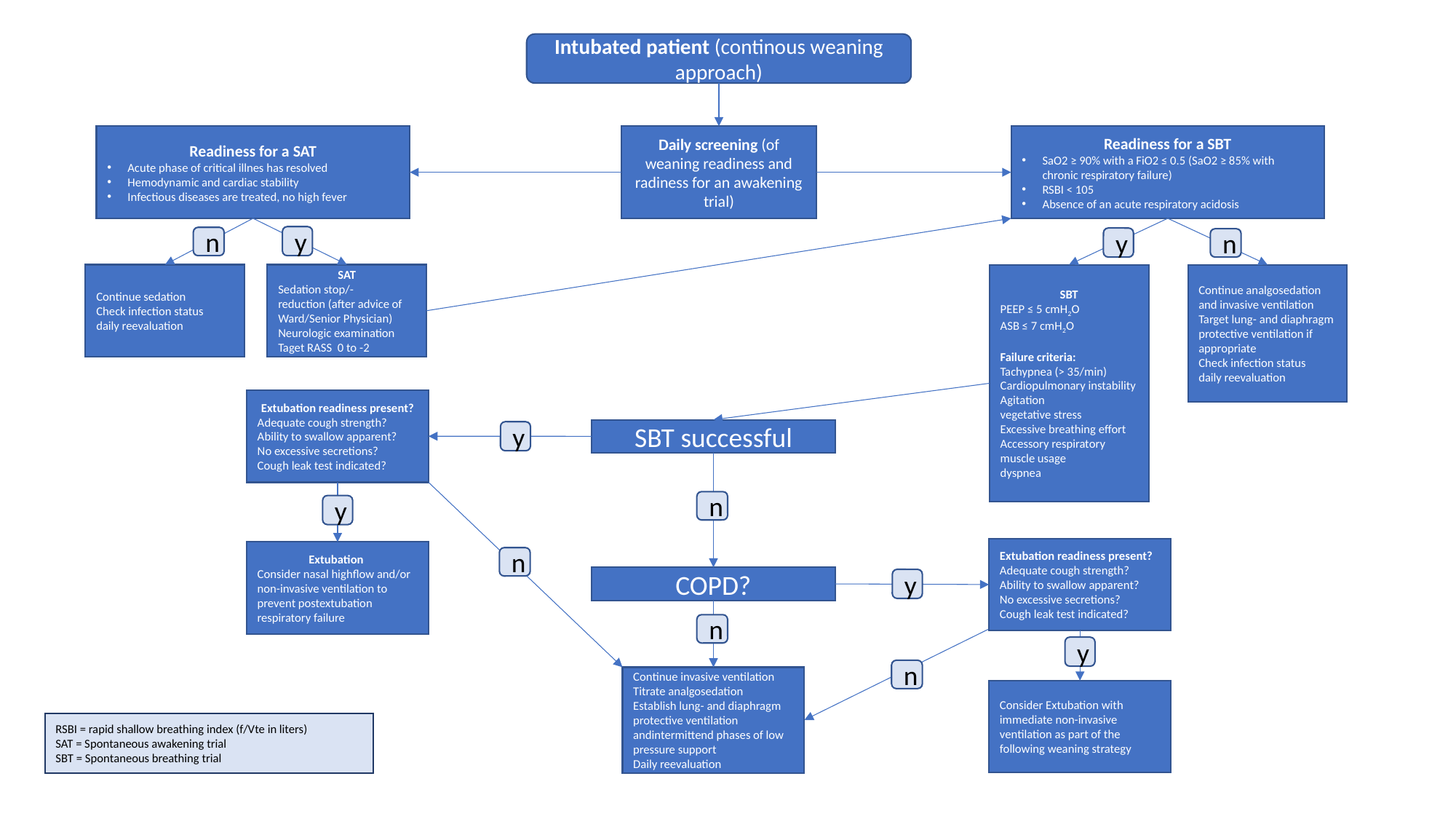

Intubated patient (continous weaning approach)
Readiness for a SBT
SaO2 ≥ 90% with a FiO2 ≤ 0.5 (SaO2 ≥ 85% with chronic respiratory failure)
RSBI < 105
Absence of an acute respiratory acidosis
Readiness for a SAT
Acute phase of critical illnes has resolved
Hemodynamic and cardiac stability
Infectious diseases are treated, no high fever
Daily screening (of weaning readiness and radiness for an awakening trial)
y
n
y
n
SBT
PEEP ≤ 5 cmH2O
ASB ≤ 7 cmH2O
Failure criteria:
Tachypnea (> 35/min)
Cardiopulmonary instability
Agitation
vegetative stress
Excessive breathing effort
Accessory respiratory muscle usage
dyspnea
Continue analgosedation and invasive ventilation
Target lung- and diaphragm protective ventilation if appropriate
Check infection status
daily reevaluation
Continue sedation
Check infection status
daily reevaluation
SAT
Sedation stop/-
reduction (after advice of
Ward/Senior Physician)
Neurologic examination
Taget RASS 0 to -2
Extubation readiness present?
Adequate cough strength?
Ability to swallow apparent?
No excessive secretions?
Cough leak test indicated?
SBT successful
y
n
y
Extubation readiness present?
Adequate cough strength?
Ability to swallow apparent?
No excessive secretions?
Cough leak test indicated?
Extubation
Consider nasal highflow and/or non-invasive ventilation to prevent postextubation respiratory failure
n
COPD?
y
n
y
n
Continue invasive ventilation
Titrate analgosedation
Establish lung- and diaphragm protective ventilation andintermittend phases of low pressure support
Daily reevaluation
Consider Extubation with immediate non-invasive ventilation as part of the following weaning strategy
RSBI = rapid shallow breathing index (f/Vte in liters)
SAT = Spontaneous awakening trial
SBT = Spontaneous breathing trial

## Slide 3
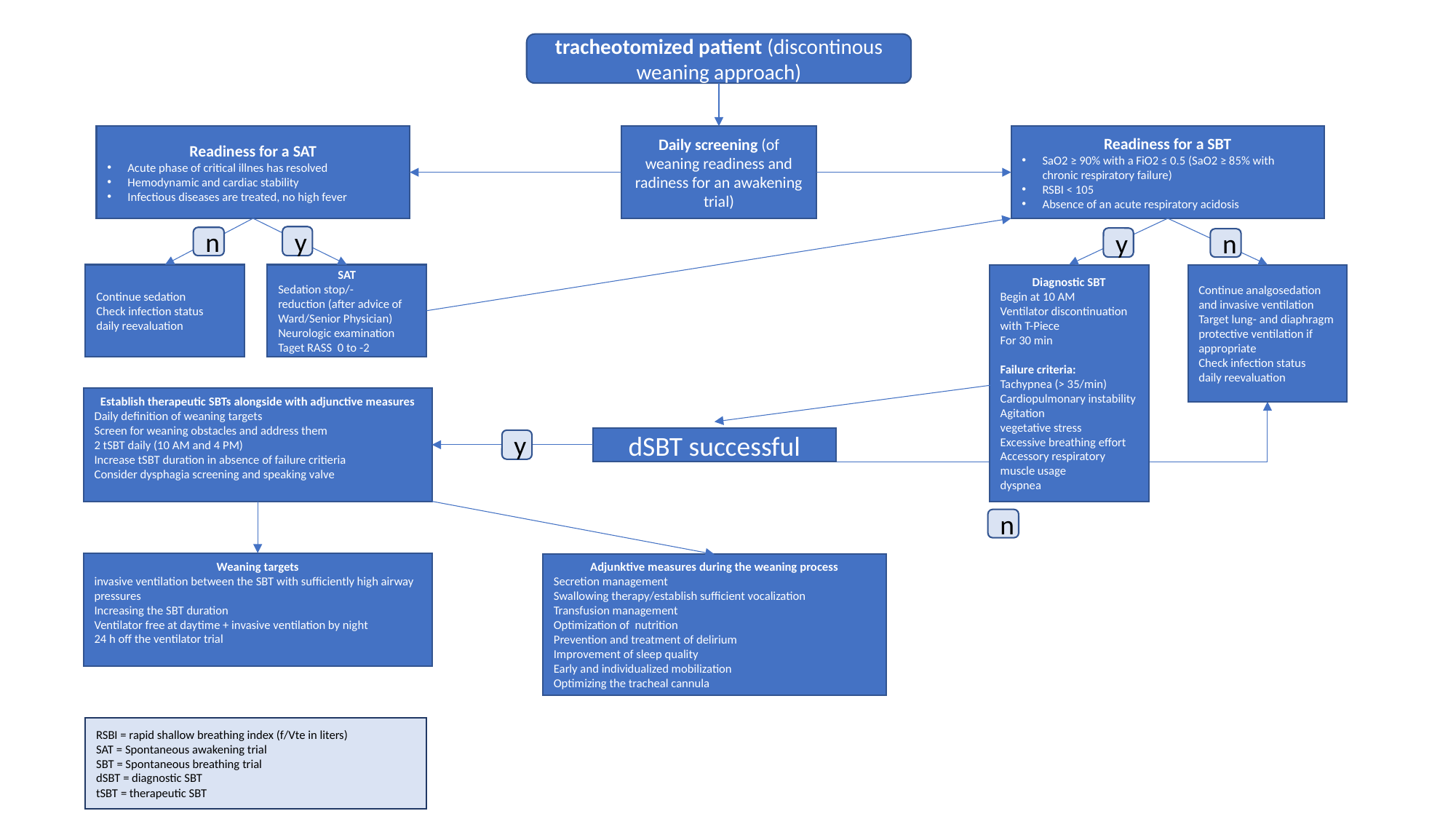

tracheotomized patient (discontinous weaning approach)
Readiness for a SBT
SaO2 ≥ 90% with a FiO2 ≤ 0.5 (SaO2 ≥ 85% with chronic respiratory failure)
RSBI < 105
Absence of an acute respiratory acidosis
Readiness for a SAT
Acute phase of critical illnes has resolved
Hemodynamic and cardiac stability
Infectious diseases are treated, no high fever
Daily screening (of weaning readiness and radiness for an awakening trial)
y
n
y
n
Diagnostic SBT
Begin at 10 AM
Ventilator discontinuation with T-Piece
For 30 min
Failure criteria:
Tachypnea (> 35/min)
Cardiopulmonary instability
Agitation
vegetative stress
Excessive breathing effort
Accessory respiratory muscle usage
dyspnea
Continue analgosedation and invasive ventilation
Target lung- and diaphragm protective ventilation if appropriate
Check infection status
daily reevaluation
Continue sedation
Check infection status
daily reevaluation
SAT
Sedation stop/-
reduction (after advice of
Ward/Senior Physician)
Neurologic examination
Taget RASS 0 to -2
Establish therapeutic SBTs alongside with adjunctive measures
Daily definition of weaning targets
Screen for weaning obstacles and address them
2 tSBT daily (10 AM and 4 PM)
Increase tSBT duration in absence of failure critieria
Consider dysphagia screening and speaking valve
dSBT successful
y
n
Weaning targets
invasive ventilation between the SBT with sufficiently high airway pressures
Increasing the SBT duration
Ventilator free at daytime + invasive ventilation by night
24 h off the ventilator trial
Adjunktive measures during the weaning process
Secretion management
Swallowing therapy/establish sufficient vocalization
Transfusion management
Optimization of nutrition
Prevention and treatment of delirium
Improvement of sleep quality
Early and individualized mobilization
Optimizing the tracheal cannula
RSBI = rapid shallow breathing index (f/Vte in liters)
SAT = Spontaneous awakening trial
SBT = Spontaneous breathing trial
dSBT = diagnostic SBT
tSBT = therapeutic SBT
